# Completely ossified thoracic intradural meningioma in an elderly patient: A case report and literature review

**DOI:** 10.1097/MD.0000000000020814

**Published:** 2020-06-19

**Authors:** Feng Xu, Zhisen Tian, Zhigang Qu, Liyu Yao, Congcong Zou, Wanrong Han, Caihong Zhang, Changfeng Fu, Yuanyi Wang

**Affiliations:** a Department of Spine Surgery, The First Hospital of Jilin University; b Department of Orthopedics, China-Japan Union Hospital affiliated to Jilin University; c Department of Pediatric Surgery, The First Hospital of Jilin University, Changchun, China.

**Keywords:** completely ossified meningioma, elderly patient, en bloc tumorectomy, post-surgical complication, thoracic spine tumor

## Abstract

**Rationale:**

Spinal meningioma is the second most common spinal neoplasm that commonly occurs in middle-aged women. As a rare histological variation of meningioma, completely ossified meningioma (OSM) and its standard surgical strategies have been reported. However, the surgical outcomes of elderly OSM cases (age >70) are less investigated. Herein, we intend to present an elderly OSM case and review the elderly OSM cases in published literatures.

**Patient concerns:**

An 85-year-old female presented with 10-year history of back pain and developed radiating pain on her left lower extremity within the last 2 weeks.

**Diagnoses:**

A magnetic resonance imaging scan revealed an intradural tumor at the T11 level. A computed tomography scan demonstrated that the mass was completely ossified and had distinct borders (Fig. 1D–F). In a dynamic contrast-enhanced magnetic resonance imaging scan, the mass presented as a lateral intradural extramedullary tumor with abundant blood supply (Fig. 2).

**Interventions:**

The patient underwent en bloc intradural tumorectomy via posterior approach.

**Outcomes:**

After surgery, the patient's pain was relieved. However, the patient spent another 3 weeks in hospital due to a series of post-surgery complications, including hypostatic pneumonia, pulmonary heart failure, hydrothorax in both thoracic cavities, hypoproteinemia, and deep venous thrombosis on both of her legs. The complications recovered after 3-weeks treatment. In 1-year follow up, no additional symptom was found and the patient was recurrence free.

**Lessons:**

Our report indicated that the surgical outcome can be satisfying in elderly OSM patients, while the post-operative complications frequently occur due to the poor physical condition of elderly patients. As a result, treatment of peri-operative complications of elderly OSM patients also deserves greater attention along with surgical resection.

## 1 Introduction

Spinal meningioma accounts for 25% to 46% of all cases of primary spine tumors.^[1]^ In terms of frequency, meningioma is the second most common neoplasm that occurs in the spine (neurilemmoma being the most common neoplasm).^[2]^ Calcification and ossification of spinal meningioma are uncommon, with an incidence of 1% to 5% among all cases of spinal meningioma.^[3]^ Most cases of meningioma occur among middle-aged women, whereas completely ossified meningioma (OSM) and calcified meningioma have been reported as rare histological variations of meningioma. OSM has been considered as an advanced form of calcified meningioma, and its diagnosis and treatment have been studied and reviewed in recent years.^[4]^ A standard surgical strategy of tumor resection has been widely accepted by clinicians.^[5]^ However, the surgical outcomes of elderly (age >70 years) cases of OSM are seldom reported. Because the ossification process occurs slowly, elderly patients with OSM also present with clinical symptoms, and subsequent surgery might lead to serious complications because of their age. Here, we present a case of a patient with OSM, who was also the second oldest patient with OSM in the world.

## 2 Case report

An 85-year-old female presented to the spine surgery clinic with a 10-year history of back pain; she had developed radiating pain on her left lower extremity within the last 2 weeks. During the physical examination, the patient was restricted in a seated position and had back tenderness and percussion. Her muscle force of both lower limbs was decreased (level 3), combined with significant sensory loss below the groin level. Her tendon reflexes were normal, and she had negative Babinski reflexes on both sides.

**Figure 1:**
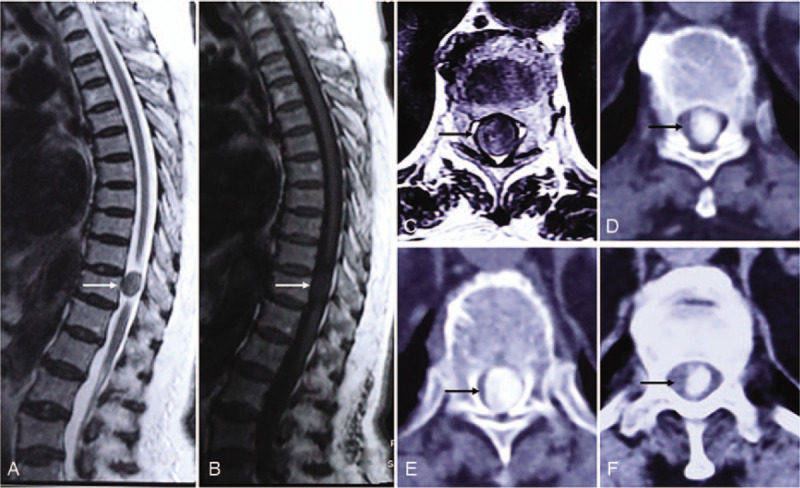
Magnetic resonance imaging revealed a mass at the T11 level. Compared to the spinal cord, the mass presented as an isointense signal on T2- and T1-weighted sequences (A and B). A computed tomography scan revealed a completely ossified mass in the spinal canal at the T11 level. The mass had clear borders and a high signal was evenly distributed within the entity (D, E, and F). The mass had a diameter that was almost equal to the inner diameter of the corresponding spinal canal, thereby causing serious compression to the nearby spinal cord (C, D, E, and F).

**Figure 2:**
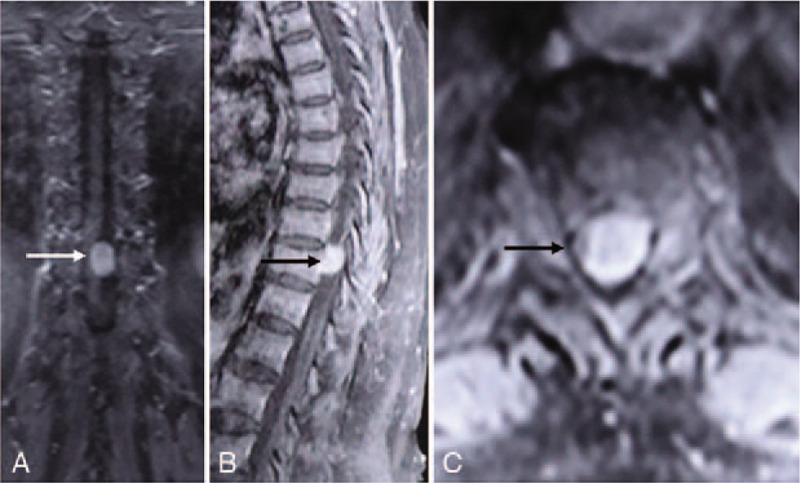
Dynamic contrast-enhanced magnetic resonance imaging scan showing a lateral intradural extramedullary tumor at the T11 level with a diffuse enhanced signal. The coronal plane revealed that the spinal cord was pushed to the right side by the mass (A); the ventral basis of the mass has unclear border with the anterior spinal canal, thus suggesting an adhesion in the area (B).

A magnetic resonance imaging (MRI) scan revealed an intradural tumor at the T11 level that caused serious compression of the thoracic spinal cord (Fig. 1A–C). A computed tomography (CT) scan demonstrated that the mass was completely ossified and had distinct borders (Fig. 1D–F). In a dynamic contrast-enhanced MRI scan, the mass presented as a lateral intradural extramedullary tumor with abundant blood supply (Fig. 2).

**Figure 3:**
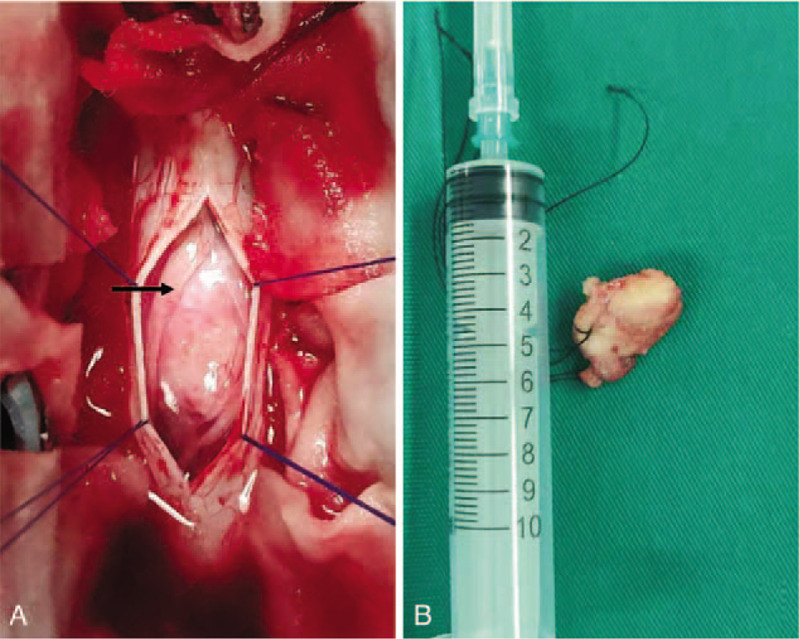
During the operation, the mass was exposed by a dura incision (A). The mass showed severe adhesion to the surrounding nerve tissues (A, arrow), and it was completely resected (B).

**Figure 4:**
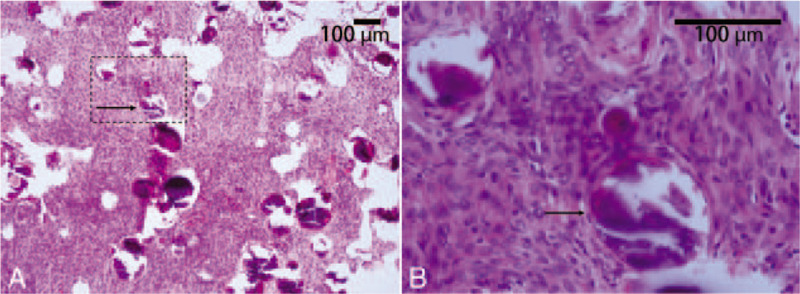
Histopathological examination (hematoxylin and eosin staining) suggested that the mass was a spinal meningioma with diffused formation of psammomatous bodies (A and B, arrow).

After pre-operative preparation, the patient underwent T9 to T12 transpedicular screw fixation, T10 to T11 laminectomy, unilateral facetectomy, and intradural tumor resection via a posterior approach. After the removal of laminar and facet elements of T10 and T11, the dura matter of the corresponding level was sufficiently exposed. A dura incision was made, and the extramedullary mass was revealed, unlike the rocky impression that was presented in the CT scan, and the lesion surface was tough and fibrotic (Fig. 3). The mass entity was closely wrapped by nerve tissues (Fig. 3), and the ventral side of the mass had serious adhesion to the arachnoid membrane and spinal cord. After the tumor base and the attached ventral arachnoid membrane were carefully separating using bipolar forceps, the mass was completely resected (Fig. 3) while the spinal cord was cautiously avoided, and the dura was closed with sutures afterwards. The operating time was 2 hours and 20 minutes, and the total blood loss was approximately 400 mL. A histopathological examination of the resected specimen revealed that the mass was a meningioma with diffused psammomatous bodies (Fig. 4).

**Figure 5:**
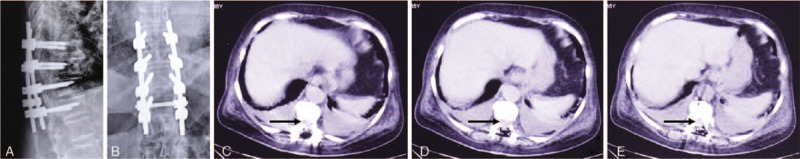
Post-surgical examinations of the patient. Digital radiography (DR) showing appropriate internal fixation (A and B). A computed tomography scan showing complete resection of the meningioma (C, D, and E, arrow); however, it also suggested hypostatic pneumonia and hydrothorax (C, D, and E).

The post-surgical examination reported complete removal of the calcified tumor and appropriate instrument location (Fig. 5). The radiating pain was relieved immediately after the surgery; however, the patient mentioned slight involuntary movement on her left lower extremity, which improved after three weeks of neurotrophic treatment. She stayed for 3 weeks in the hospital due to the perioperative complications. Chronologically, the patient had cerebral fluid (CF) leakage (300 mL/d) due to the absence of biomaterial product that could reinforces the dura suture. During the period of bed stay without pillow, which prevented severe CF leakage, she further developed hypostatic pneumonia, which leaded to pulmonary heart failure and hydrothorax in both thoracic cavities. Moreover, she had hypoproteinemia, and deep venous thrombosis (DVT) in both of her legs because of albumin loss and chronic consumption and long-time bed stay. She received antibiotic therapy (cefodizime, 20 mg/BID), sputum excretion, thoracic drainage, and anti-thrombosis therapy and was administered a protein-rich diet and albumin intravenous drip. After 3 weeks, her blood protein level increased, and the hydrothorax and cardio-related symptoms were subsequently relieved. Then, her DVT transformed to chronic thrombus, and she was discharged from the hospital three weeks post-surgery. During the 1-year follow-up, the patient did not have any additional symptoms and was recurrence-free.

## 3 Discussion

Including the current case, a total of 10 cases of elderly (age > 70 years) patients with OSM have been published up till 2019 according to PubMed (Table 1). The average age was 78 years, and our patient is the second oldest patient who underwent OSM tumorectomy. According to the published case reports, the predominant incidences among females have been clearly noted among elderly patients (9 women and 1 man), and most cases of OSMs (9 cases) occurred in the thoracic spine, with only one exception that occurred at L3. Interestingly, most patients did not report a symptomatic history over 2 years, and several cases remained asymptomatic until an event such as falling occurred. According to the onset peak and age range of the cohort, the mass was speculated to have been asymptomatic or slightly symptomatic for a long time; the mass then became unstable under stimulation, which might result in bleeding, displacement, and fracture of the mass, thereby shifting and causing nerve compression and aggravated nervous symptoms. For treatment, all of the reviewed cases underwent tumor resection, in which the tumor was removed in 6 cases via total en bloc resection, and 3 cases’ tumor was removed with adherent dura matter. All of the patients showed immediate or gradual improvement after surgery, and no recurrence was noted in the follow-ups.

**Table 1:**
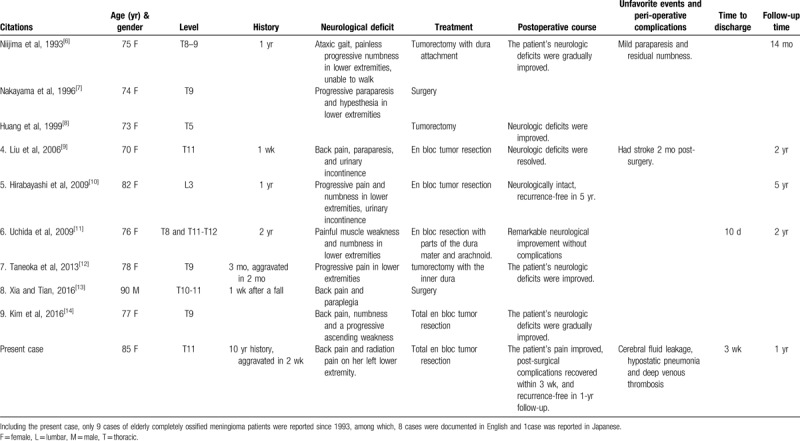
Summary of all reported cases of elderly (age > 70 yr) patients with completely ossified meningioma.

Calcifications in cases of spine meningioma are rare, and gross calcification was only observed in 1% to 5% of all cases of spinal meningioma.^[3]^ Cases of completely ossified spinal meningioma are even rarer. The classification by the World Health Organization (WHO) states that the histological classification of ossified meningioma has been categorized as a phenotype of meningioma with mesenchymal component expression.^[15]^ Based on the calcification degree of meningioma, Ruggeri et al proposed a classification that included 3 subgroups: micro-calcified, macro-calcified, and ossified. In Ruggeri's classification, meningiomas that only have histological evidence of calcification are categorized as micro-calcified; macro-calcified meningiomas and ossified meningiomas refer to a mass that has gross calcification in part or in total, respectively, which can be identified in a CT scan.^[16]^ This classification clarified the distinction of calcification and ossification of meningioma.

Currently, researchers agree that meningioma is a form of dysplasia that arises from arachnoid cap cells originating from the outer layer of the arachnoid mater and villi^[2]^; however, the pathogenesis of OSM is still controversial. Based on the histological presentation of calcified meningioma, WHO has described three common histological subtypes of OSM, including transitional, psammomatous, and metaplastic.^[17]^ In a study by Kubota et al, the progression of psammomatous body formation was described. According to their theory, ossification was presented as an advanced and large calcification of the psammomatous body with the progressive accumulation of a hydroxyapatite crystal,^[18]^ which explained the formation of the psammomatous body in our case. Another study proposed that the ossification started from metaplasia of arachnoid cells including premature arachnoidal cells and meningothelial cells, which induce the synergistic effect of osteoblast, fibroblast, and angiogenesis components in bone tissue formation, thus promoting the ossification in meningioma.^[19]^ Uchida et al suggested that the calcification and ossification progress is induced by the activation of the ossification cascade or exposure to osteoblast transforming factors including SOX9 and Runx-2.^[11]^ However, along with the findings of bone histogenesis and bone tissue in cases of OSM,^[2,8]^ researchers have begun to realize that the metaplastic differentiation is also critical in all subtypes.

The diagnosis of calcification and ossification meningioma is not difficult since the lesion sometimes can be observed in a digital radiograph (DR), and more easily identified in a CT scan, which is also the sorting method of macro-calcified meningioma and ossified meningioma.^[16]^ MRI is helpful when evaluating the anatomical localization and neurovascular involvement of neurogenic tumors including meningioma. Usually, OSM appears as hypo- or isointense signals in the spinal cord on T1- and T2-weighted sequences with hypointense signals in calcified areas. Furthermore, the OSM signal can be enhanced by contrast, which requires a differential diagnose apart from other tumors including schwannoma, lymphoma, and metastasis.^[20,21]^ However, MRI is not as sensitive as CT in detecting small amounts of calcification, and the uncertain signal (mixed or high density^[6]^) of calcification presented in a T1-weighted sequence further increases the difficulty in identifying calcifications via MRI. Therefore, CT has been considered as an important tool in OSM to delineate the location and margin of the mass.^[7]^

For benign meningioma, surgery is the primary treatment for OSM, and a satisfying outcome can be expected when the mass is completely removed. However, unlike typical spinal meningiomas, OSM usually adheres to surrounding nerve tissues. The pattern of adhesion might be attributed to its scant cellularity consisting of acellular concretions.^[21]^ Especially for elderly patients, the lack of a clear dissection plane due to the metaplasia of surrounding arachnoid membrane might result in surgical difficulties and risks that lead to worse surgical outcome compared to usual meningiomas.^[22]^ Thus, the prime goal of surgery is furthest tumor resection with careful maintenance of nerve tissue.^[2]^ In our experience, OSM resection for elderly patients is performed with several principles. First, a thorough and detailed pre-surgical preparation is necessary. The common underlying diseases of the circulation and respiration systems should be properly treated until the patient is able to tolerate general anesthesia. The mass's location and size should be fully estimated, and the minimal range of laminectomy must be discussed by the surgical group. Secondly, the duration of the surgery must be minimal, which results in less anesthetic risks and blood loss. Thirdly, considering the age and recurrent rate of OSM among elderly patient, if the base of the OSM closely adheres to the surrounding nerve tissues, which requires a rather long time to resect it entirely, the base adhesion is better coagulated or directly debulked with the attached inner dura layer than separating the adhesion, thereby reducing the risks of anesthesia and nerve injury. At last, the dura matter has to be carefully closed to prevent serious cerebral leakage after the surgery.

According to published studies, favorable surgical and satisfying follow-up outcomes have been reported (Table 1); nonetheless, post-surgical complications of elderly patients with OSM have been insufficiently documented. The perioperative complications are seldomly referred among the reported elderly OSM cases, only 1 case was reported that suffered stroke after operation^[9]^ and residual numbness and paraparesis were as well documented. The capacities of the major systems in elderly patients is rather lower than that of younger patients, and their physical condition is usually weakened by surgery and long-term bed-stay. Thus, as to elderly patients with neurologic deficits and recently underwent intradural surgery, complications including hypostatic pneumonia, pulmonary-related heart failure, DVT, and hypoproteinemia-related symptoms such as edema and delayed incision healing are more likely to happen. The occurrence of post-surgical complications in elderly patients is characterized by the “fuse” effect, which means that 1 complication is able to lead to several subsequent complications in other systems. In the present case, post-operative CF leakage exaggerated the perioperative complications that were resulted from the patient's poor physical condition and surgery strike. She was required to stay in bed for 1 week due to cerebral fluid leakage caused by the dura incision, which led to hypostatic pneumonia and DVT formation. Moreover, the cerebral fluid leakage and hypoproteinemia worsened her inner osmotic pressure, which subsequently induced the hydrothorax and swelling in her lower extremities. Thus, effective suture of the dura matter to prevent serious leakage of cerebral fluid is necessary for elderly patients, which can effectively shorten the bed-rest duration and allow for early off-bed movement and can also help to prevent hypostatic pneumonia, DVT, and calcium loss.

## 4 Conclusions

We presented a rare case of a patient with OSM, who was also the second oldest patient that underwent OSM resection. However, due to the elderly patient's poor physical condition, she developed post-surgical complications in multi-systems. Through this case, we summarized that performing intradural surgeries to elderly patients required well-designed surgical strategy, short surgery time, perfect closing of dura matter, and pre-operative physical preparation. Once the post-surgical complications occur, it is vital to “cut the fuse”. As longtime bedrest is the main cause of several post-surgical complications in elderly patients, early off-bed movement is recommended. Along with the precise surgical resection of OSM, peri-operative treatment of elderly OSM patients also deserves greater attention.

## Author contributions

**Conceptualization:** Changfeng Fu, Yuanyi Wang.

**Funding acquisition:** Yuanyi Wang.

**Investigation:** Changfeng Fu.

**Project administration:** Yuanyi Wang.

**Resources:** Feng Xu, Congcong Zou, Wanrong Han.

**Software:** Zhisen Tian, Zhigang Qu, Liyu Yao.

**Supervision:** Yuanyi Wang.

**Validation:** Caihong Zhang.

**Visualization:** Zhisen Tian, Liyu Yao, Caihong Zhang.

**Writing – original draft:** Feng Xu, Zhisen Tian, Yuanyi Wang.

**Writing – review & editing:** Zhigang Qu, Yuanyi Wang.
